# Factor structures and psychometric properties of three brief versions of the difficulties in emotion regulation scale in the Korean population

**DOI:** 10.1186/s40359-024-02261-z

**Published:** 2024-12-18

**Authors:** Gyumyoung Kim, Minkyung Yim, Hayoung Bae, Ji-Won Hur

**Affiliations:** School of Psychology, Korea University, Seoul, Korea

**Keywords:** DERS, Emotion dysregulation, Bifactor model, Brief form, Validation

## Abstract

**Background:**

This study examined the latent factor structures and psychometric properties of three brief versions of the Difficulties in Emotion Regulation Scale (DERS)—DERS-SF, DERS-18, and DERS-16—across large-scale samples of the Korean population.

**Methods:**

Participants from two independent community samples (*N* = 862 and *N* = 1,242) completed an online self-report survey, including brief versions of the DERS and associated measures. Confirmatory factor analyses were employed to examine the latent factor structures of the brief versions of the DERS with comparable models. The internal consistency, concurrent validity, and convergent validity of the brief versions of the DERS were also assessed.

**Results:**

The findings revealed that the bifactor models of the DERS-SF and DERS-18, excluding the Awareness subscale, showed superior fit indices for latent factor structure and favorable reliability. By contrast, the DERS-16 exhibited inadequate fit. Scores from the DERS-SF and DERS-18 demonstrated significant associations with indicators of psychological distress, supporting their convergent validity. The Awareness subscale showed lower internal consistency and distinctive correlation patterns with clinical outcomes.

**Conclusions:**

These findings highlight the robustness of the DERS-SF and DERS-18 as parsimonious and efficient measures of emotion dysregulation with fewer items than the original version. Furthermore, this study provides additional support for excluding the Awareness subscale when using the brief versions of the DERS.

## Introduction

Emotion regulation refers to the conscious or unconscious process of modulating one’s emotional experiences [1]. Difficulties in emotion regulation have been considered a transdiagnostic factor for various psychiatric disorders and symptoms [2–4] including depression [5], bipolar disorder [6], substance use disorder [7], eating disorder [8], borderline personality disorder (BPD) [9], posttraumatic stress disorder [10], social anxiety disorder [11], and autism spectrum disorder [12]. These difficulties in emotion regulation have also been linked to maladaptive coping strategies such as nonsuicidal self-injury [13–15] and substance misuse [16]. A recent meta-analysis found that emotion regulation significantly impacts not only the development of mental disorders, but also the well-being of individuals with mental disorders [17].

The Difficulties in Emotion Regulation Scale (DERS), developed by Gratz and Roemer [18], serves as a primary measure for evaluating emotion dysregulation. This 36-item self-report questionnaire comprehensively assesses multidimensional aspects of emotion dysregulation, including limited awareness of emotional responses (Awareness), lack of a clear understanding of emotional responses (Clarity), difficulty engaging in goal-directed behavior during negative emotional experiences (Goals), challenges in impulse control during negative emotional experiences (Impulse), nonacceptance of emotional responses (Nonacceptance), and limited access to effective emotion regulation strategies (Strategies). Numerous studies have established the reliability and validity of the DERS across diverse populations. These include community samples of adolescents [19] and adults [20], and clinical samples such as psychiatric inpatients [21] and adults with emotional disorders [22]. In addition, a large number of studies have employed the DERS to assess treatment efficacy in addressing emotion dysregulation in various clinical trials involving cognitive behavioral therapy, dialectical behavior therapy, and emotion regulation therapy [23–25].

In its initial development, the DERS was conceptualized as a multidimensional, correlated six-factor model, which has also been confirmed in the prior literature as the best factor structure for the DERS [26, 27]. However, a growing number of recent studies support the notion that the DERS is best represented by the bifactor model [22, 28], which is emerging as a promising candidate for an optimal factor structure of the scale. The bifactor and correlated multi-factor model both capture the multidimensionality of the DERS. However, while the correlated multi-factor model evaluates multiple latent dimensions that independently influence a set of items, the bifactor model provides a framework for examining whether an instrument consists of a general factor that accounts for shared variance across domains (difficulties in emotion regulation), alongside specific factors that capture unique variance within each domain (each subscale of the DERS). This provides guidelines for determining the adequacy of computing a total score of the DERS and using different subscales. Therefore, exploring the most appropriate latent factor structure of the DERS in diverse populations has significant implications for improving clinical research and interventions in emotion regulation.

Another consideration involves the psychometric properties of the Awareness subscale. For example, previous studies have shown lower correlations of the Awareness subscale with both the DERS total score and other subscales [22, 29]. This has prompted questions about whether the Awareness subscale measures the same emotion dysregulation construct as other subscales. Moreover, the Awareness subscale has exhibited inconsistent relationships with clinical outcomes related to emotion regulation, such as a lack of significant correlation with the severity of BPD symptoms [29]. Prior findings have also revealed inverse or nonsignificant correlations between the Awareness subscale and psychological distress such as depression, anxiety, and stress [22, 30].

Therefore, the inclusion of the Awareness subscale has been intensively examined in recent studies validating the factor structure of the DERS, resulting in some research supporting the original six-factor structure of the scale [14, 19, 20]. By contrast, other studies have proposed a five-factor structure of the DERS that excludes the Awareness subscale [22, 29, 31, 32]. Alternative versions of the DERS with a modified Awareness subscale have also been suggested [33, 34]. Notably, determining whether the Awareness subscale adequately loads onto the general emotion dysregulation factor is an important issue in the bifactor model. Several pieces of evidence suggested that DERS is best represented by the bifactor model that excludes the Awareness subscale [22, 28]. However, further investigation is required to determine whether a correlated five-factor model or a bifactor model excluding the Awareness scale is more appropriate.

The brief versions of the DERS, including the DERS-Short Form (DERS-SF) [35], DERS-18 [36], and DERS-16 [37], were developed to more efficiently measure emotion dysregulation among larger samples. The psychometric properties of these brief versions have been confirmed in community samples [38, 39] and diverse, culturally broader samples [28, 30, 40]. However, there is no consensus on the optimal factor structure for each version [22, 38, 39, 41]. To date, there have been no direct comparative studies on the psychometric properties and different factor solutions of the brief versions of the DERS in the same large sample of the general population.

Therefore, in this study, we sought to evaluate the factor structures and psychometric properties of three brief versions of the DERS in large-scale community samples of the Korean population. This research comprises two separate studies (Study 1 and Study 2) with distinct samples to enhance the replicability and generalizability of the findings. In Study 1, we preliminarily examined the factor structures of three brief versions of the DERS in a large sample of young adults from the general population. Concurrent validity was assessed by examining the correlations between these brief versions and the original DERS. In Study 2, we evaluated the factor structures and psychometric properties of the brief versions of the DERS that demonstrated adequate model fit in Study 1. A large sample of adults from the general population was used to a) re-examine the factor structure, b) examine its internal consistency, and c) assess convergent validity. This type of comparative study could provide practical insights into factor structures and the psychometric properties of these brief versions of the DERS, thus aiding researchers and clinicians in determining the most appropriate measures and analytical approaches for assessing emotion dysregulation.

## Methods

### Participants and data collection procedures

For Study 1, the sample of participants (*N* = 862*, M*_age_ = 22.72, *SD* = 2.84) was derived from a more extensive investigation of emotional distress in emerging adults aged 19 to 29 years. Participants with fluency in written Korean were recruited through online advertisements. Of the 911 participants who completed the survey, 49 were excluded from the analysis because they did not meet the age criteria. Among the included participants, 72.4% (*n* = 624) were female. The entire survey took approximately 30 to 40 min to complete. The original 36-item DERS was analyzed in this study, and each brief version of the DERS was recalculated based on responses to the original 36-item DERS. The Institutional Review Board of Korea University (KUIRB) approved Study 1 (KUIRB-2020–0079-02; 2021–0187-02). All participants in Study 1 completed informed consent forms before the survey.

The sample for Study 2 (*N* = 1,242; *M*_age_ = 24.43, *SD* = 4.74) was drawn from a mental health survey dataset administered by a university counseling center. Students were invited to participate in the survey via email. Those who voluntarily chose to participate received a link to the online survey with a consent form from the university counseling center. The consent form included a statement that the data provided by participants could be processed and used for research purposes in the future after eliminating any information that could identify participants. Of these participants, 59.7% (*n* = 742) were female, 40.0% were male (*n* = 497), and three reported a transgender/gender non-conforming identity. Informed consent for participants in Study 2 was waived by the KUIRB as the dataset did not contain any direct identifiers of participants (KUIRB-2023–0214-01).

### Instruments

#### Difficulties in emotion regulation scale

The original DERS [18] is a 36-item self-report questionnaire scored on a five-point Likert scale ranging from 1 to 5. It assesses six dimensions of emotion dysregulation: Awareness, Clarity, Goals, Impulse, Nonacceptance, and Strategies. The validation study of the Korean version of the original 36-item DERS showed a six-factor structure [42]. The modified five-factor model, which integrated the Awareness and Clarity subscales into the Understanding subscale, controlling for the method factor, has also been proposed [33]. The overall internal consistency of the DERS observed in Study 1 was high (Cronbach’s α = 0.95, McDonald’s ω = 0.96).

#### Brief versions of the difficulties in emotion regulation scale

The psychometric properties of three brief versions of the DERS were examined. The first brief version, the DERS-16, was developed by considering item-total correlations and content validity [37]. In the development of the DERS-16, the Awareness subscale was removed because of its lower correlations with other subscales. To create the second brief version, DERS-SF, items were selected based on the outcomes of exploratory factor analyses in previous research on the original 36-item DERS [35]. Confirmatory factor analyses (CFA) were subsequently conducted among adolescents and adults. Finally, the formulation of the DERS-18 involved selecting three items with the highest factor loadings from each of the six factors [36]. The psychometric properties of the DERS-18 were examined across five different samples, varying in age and sample type, to ensure robustness. The Awareness subscale was retained in both the DERS-SF and DERS-18.

#### Center for epidemiologic studies depression scale revised – 10

The Center for Epidemiologic Studies Depression Scale Revised (CESD-R-10) is the shortened version of the Center for Epidemiologic Studies Depression Scale Revised (CESD-R) [43, 44]. It measures the affective and somatic components of depression. Each of the 10 items is scored on a five-point Likert scale. The Korean version of the CESD-R-10 [45] used in this study demonstrated acceptable internal consistency (Cronbach’s α = 0.88, McDonald’s ω = 0.89).

#### Generalized anxiety disorder – 7

The Generalized Anxiety Disorder – 7 (GAD-7) comprises seven items reflecting the most prominent diagnostic features of generalized anxiety disorder [46]. Respondents are asked to indicate how often they have been bothered by symptoms of anxiety in the past two weeks. Each item is scored on a four-point Likert scale ranging from 0 (not at all) to 3 (almost every day). The Korean version of the GAD-7 demonstrated good internal consistency in this study (Cronbach’s α and McDonald’s ω = 0.90).

#### Perceived stress scale—10

The Perceived Stress Scale (PSS) assesses how stressful a respondent perceives their life to be over the past month based on 14 items measured on a five-point Likert scale [47]. As previous research has indicated that the 10-item version has better internal consistency [48], the PSS-10 version was used in this study. The internal consistency in this study was within the acceptable range (Cronbach’s α and McDonald’s ω = 0.86).

#### Self-compassion scale, short form

The Self-Compassion Scale (SCS) measures six components of self-compassion—Self-Kindness, Self-Judgment, Common Humanity, Isolation, Mindfulness, and Over-Identification—on a five-point Likert scale [49]. The 12-item short form of the SCS (SCS-SF) used in this study [50] demonstrated acceptable internal consistency (Cronbach’s α and McDonald’s ω = 0.88).

### Data analysis

Missing data were not imputed as there were no missing data in either sample. Demographics, internal consistency, and Pearson’s correlation coefficients were analyzed using R version 4.3.1. Both Cronbach’s α and McDonald’s ω were used to assess the internal consistency of the questionnaires used in this study, because Cronbach’s α may underestimate true reliability when the tau-equivalence is violated [51]. Internal consistency indices were considered acceptable if they were greater than 0.7.

In Study 1, we conducted CFAs to examine the factor structures of the DERS brief versions. Mplus version 8.8. was used for all CFAs. Five models were examined for the DERS-SF and DERS-18 to evaluate the latent factor structure and determine whether the Awareness subscale should be included: 1) correlated six-factor model; 2) correlated five-factor model; 3) bifactor model including Awareness; 4) bifactor model excluding Awareness; and 5) modified five-factor model, which integrated the Awareness and Clarity subscales into the Understanding subscale. Goodness-of-fit was assessed using the comparative fit index (CFI), standardized root mean squared residuals (SRMR), and root mean squared error of approximation (RMSEA). Adequate model fit is indicated by a CFI value of greater than 0.95 [52, 53], SRMR values between zero and 0.08 [53], and RMSEA values less than 0.08 [54].

In Study 2, we re-examined the latent factor structures of the DERS brief versions in another sample by performing CFAs. To evaluate dimensionality and the reliability of total and subscale scores of the bifactor models, the examined explained common variance (ECV) [55], omega hierarchical (ω_H_) [56], and omega hierarchical subscale (ω_HS_) for the corresponding specific factors [56] were computed. In this study, an ECV value greater than 0.70 [56] and an ω_H_ value greater than 0.80 [56] were considered indicative of unidimensionality for the total score. In contrast, ω_HS_ values greater than 0.50 were deemed sufficient to interpret subscale scores as meaningful [57]. In addition, we assessed internal consistency and convergent validity by examining the association between the DERS brief versions and measures of depressive symptoms (CESD-R-10), anxiety (GAD-7), perceived stress (PSS-10), and self-compassion (SCS-SF).

## Results

### Study 1

#### Construct validity

Table 1 shows the model fit indices of each DERS brief version in Study 1.

**Table 1:** Study 1: Results of the confirmatory factor analyses of each brief version of the DERS (*N* = 862)

	**χ**^**2**^	***df***	**RMSEA**	**CFI**	**SRMR**
**DERS-16**	818.399	94	0.095	0.928	0.046
**DERS-SF**					
Correlated multi-factor model					
Model 1 (Correlated six-factor model)	667.892	120	0.073	0.941	0.051
Model 2 (Correlated five-factor model)	460.533	80	0.074	0.955	0.044
Bifactor model					
Model 3 (Bifactor model including Awareness)	709.917	117	0.077	0.936	0.061
Model 4 (Bifactor model excluding Awareness)	404.006	75	0.071	0.961	0.042
Modified model					
Model 5 (Modified five-factor model)	667.709	122	0.072	0.941	0.057
**DERS-18**					
Correlated multi-factor model					
Model 6 (Correlated six-factor model)	700.479	120	0.075	0.940	0.054
Model 7 (Correlated five-factor model)	504.917	80	0.078	0.952	0.047
Bifactor model					
Model 8 (Bifactor model including Awareness)	700.124	117	0.076	0.940	0.060
Model 9 (Bifactor model excluding Awareness)	409.667	75	0.072	0.962	0.039
Modified model					
Model 10 (Modified five-factor model)	695.416	122	0.074	0.941	0.060

*DERS* Difficulties in Emotion Regulation Scale, *SF* Short Form, *df* Degrees of freedom, *RMSEA* Root mean squared error of approximation, *CFI* Comparative fit index, *SRMR* Standardized root mean squared residuals

### DERS-16

The DERS-16 showed an inadequate model fit, with a CFI of less than 0.95 and RMSEA of greater than 0.08 (*χ*^2^ = 818.399 [*df* = 94]; RMSEA = 0.095; CFI = 0.928; SRMR = 0.046). Therefore, it was excluded from Study 2.

### DERS-SF

To assess the construct validity of the DERS-SF, five models were examined: the correlated six-factor model (Model 1); correlated five-factor model (Model 2); bifactor model including the Awareness subscale (Model 3); bifactor model excluding the Awareness subscale (Model 4); and modified five-factor model (Model 5), which integrates the Awareness and Clarity subscales into the Understanding subscale.

Among these models, Model 4, the bifactor model excluding the Awareness subscale, demonstrated the most acceptable model fit indices (*χ*^2^ = 404.006 [*df* = 75]; RMSEA = 0.071, CFI = 0.961; SRMR = 0.042). In terms of factor structure, the models excluding the Awareness subscale showed better model fit indices than those including this subscale, as indicated by the lower SRMR values (Model 1, SRMR = 0.051; Model 2, SRMR = 0.044; Model 3, SRMR = 0.061; Model 4, SRMR = 0.042) and higher CFI values (Model 1, CFI = 0.941; Model 2, CFI = 0.955; Model 3, CFI = 0.936; Model 4, CFI = 0.961). Although the RMSEA value was slightly lower in Model 1 than in Model 2, the difference was small (Model 1, RMSEA = 0.073; Model 2, RMSEA = 0.074). Finally, Model 5 exhibited marginally acceptable model fit indices, with a CFI of less than 0.95 (*χ*^2^ = 667.709 [*df* = 122]; RMSEA = 0.072, CFI = 0.941; SRMR = 0.057).

### DERS-18

To assess the structural validity of the DERS-18, all five aforementioned models were tested in this study (Model 6 through Model 10). Similar to the DERS-SF, the bifactor model excluding the Awareness subscale exhibited the best model fit (Model 9, *χ*^2^ = 409.667 [*df* = 75]; RMSEA = 0.072; CFI = 0.962; SRMR = 0.039). The five-factor models generally showed a better model fit than the six-factor models, as indicated by lower SRMR values (Model 6, SRMR = 0.054; Model 7, SRMR = 0.047; Model 8, SRMR = 0.060; Model 9, SRMR = 0.039) and higher CFI values (Model 6, CFI = 0.940; Model 7, CFI = 0.952; Model 8, CFI = 0.940; Model 9, CFI = 0.962). The RMSEA value was lower in the correlated six-factor model (Model 6, RMSEA = 0.075) than in the correlated five-factor model (Model 7, RMSEA = 0.078), but the difference was very small. Finally, the model fit indices of the modified five-factor model of the DERS were relatively poor compared with the five-factor model or bifactor model excluding the Awareness subscale (Model 10, *χ*^2^ = 695.416 [*df* = 122]; RMSEA = 0.074; CFI = 0.941; SRMR = 0.060).

As an interim summary, after reviewing the model fits for all brief versions of the DERS in this study, the DERS-SF and DERS-18 performed better than the DERS-16 in the Korean population. In addition, the five-factor models, excluding the Awareness subscale, generally performed better than the six-factor models.

#### Internal consistency

Table 2 summarizes the internal consistency of each DERS brief version from Study 1. Internal consistency was acceptable for all three DERS brief versions for the Clarity, Goals, Impulse, Nonacceptance, and Strategies subscales (Cronbach’s α: 0.77 to 0.90, McDonald’s ω_S_ for each subscale: 0.78 to 0.90). However, the Awareness subscale, which is only included in the DERS-SF and DERS-18, demonstrated lower internal consistency (Cronbach’s α = 0.66 in the DERS-SF and 0.69 in the DERS-18; McDonald’s ω_S_ = 0.69 in the DERS-SF and 0.72 in the DERS-18).

**Table 2:** Internal consistency and concurrent validity of the DERS-16, DERS-SF, and DERS-18 (*N* = 862)

Scales	DERS-16			DERS-SF			DERS-18		
	**α**	**ω / ω**_**S**_	***r***	**α**	**ω / ω**_**S**_	***r***	**α**	**ω / ω**_**S**_	***r***
Overall—Six factors	-	-	-	0.92	0.92	0.99	0.92	0.92	0.98
Overall—Five factors	0.94	0.94	0.97	0.93	0.93	0.98	0.93	0.93	0.98
Awareness	-	-	-	0.66	0.69	0.83	0.69	0.72	0.81
Clarity	0.79	0.79	0.89	0.83	0.84	0.94	0.83	0.84	0.94
Goals	0.88	0.88	0.97	0.90	0.90	0.97	0.90	0.90	0.97
Impulse	0.90	0.90	0.96	0.89	0.89	0.96	0.89	0.89	0.96
Nonacceptance	0.85	0.85	0.96	0.84	0.85	0.97	0.86	0.86	0.95
Strategies	0.86	0.86	0.97	0.77	0.78	0.95	0.83	0.84	0.93

*DERS* Difficulties in Emotion Regulation Scale, *SF* Short Form, *r* = Pearson’s correlations between each subscale (or total score) and its corresponding original DERS subscale (or the DERS total scores). All correlations are statistically significant at *P* < .001

#### Concurrent validity

To assess concurrent validity, Pearson’s correlation coefficients were calculated between each subscale of all three brief versions and the original DERS (Table 1). All scales showed significant positive correlations of 0.90 or higher, except for the Clarity subscale of the DERS-16 and the Awareness subscale of the DERS-SF and DERS-18, which had relatively low correlations of 0.83 and 0.81, respectively.

### Study 2

In Study 2, construct validity, internal consistency, and convergent validity were examined for the DERS-SF and DERS-18. To examine dimensionality, the ECV, ω_H_, and ω_HS_ values for the bifactor models of the DERS-SF and DERS-18 were calculated. The DERS-16, which had a poor model fit, was excluded from this study.

#### Construct validity

Table 3 shows the model fit indices of each DERS brief version in Study 2. Analyses were conducted on each of the five models of the DERS-SF and DERS-18 (Model 11 through Model 20) to determine which model best measured the latent factor structure of the brief versions of the DERS.

**Table 3:** Study 2: Results of the confirmatory factor analysis of the DERS-SF and DERS-18 (*N* = 1,242)

	**χ**^**2**^	***df***	**RMSEA**	**CFI**	**SRMR**
**DERS-SF**					
Correlated multi-factor model					
Model 11 (Correlated six-factor model)	912.660	120	0.073	0.942	0.066
Model 12 (Correlated five-factor model)	531.846	80	0.067	0.962	0.040
Bifactor model					
Model 13 (Bifactor model including Awareness)	813.797	117	0.069	0.949	0.059
Model 14 (Bifactor model excluding Awareness)	420.056	75	0.061	0.971	0.038
Modified model					
Model 15 (Modified five-factor model)	867.522	122	0.070	0.945	0.057
**DERS-18**					
Correlated multi-factor model					
Model 16 (Correlated six-factor model)	1064.485	120	0.080	0.933	0.070
Model 17 (Correlated five-factor model)	686.465	80	0.078	0.952	0.051
Bifactor model					
Model 18 (Bifactor model including Awareness)	864.663	117	0.072	0.947	0.059
Model 19 (Bifactor model excluding Awareness)	471.030	75	0.065	0.968	0.038
Modified model					
Model 20 (Modified five-factor model)	1013.552	122	0.077	0.937	0.062

*DERS* Difficulties in Emotion Regulation Scale, *SF* Short Form, *df* Degrees of freedom, *RMSEA* Root mean squared error of approximation, *CFI* Comparative fit index, *SRMR* Standardized root mean squared residuals

**Fig. 1:**
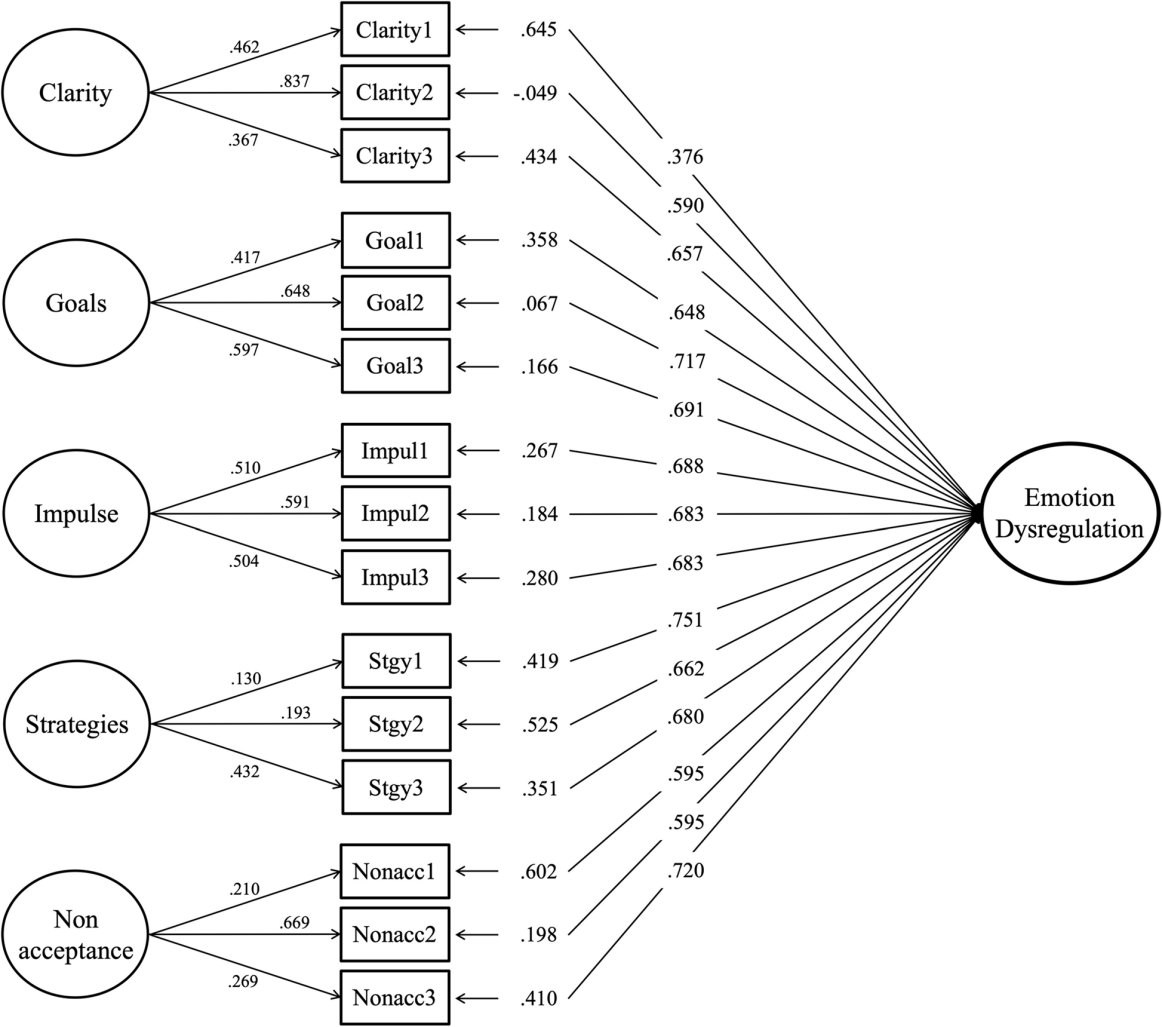
Path diagram of the DERS-SF bifactor model excluding the awareness. Note. DERS, Difficulties in Emotion Regulation Scale; SF, Short Form

Similar to Study 1, the bifactor model excluding the Awareness subscale showed the best model fit indices among other factor structures for both the DERS-SF (Model 14, *χ*^2^ = 420.056 [*df* = 75]; RMSEA = 0.061; CFI = 0.971; SRMR = 0.038) and DERS-18 (Model 19, χ^2^ = 471.030 [*df* = 75]; RMSEA = 0.065; CFI = 0.968; SRMR = 0.038). Standardized factor loadings for the Model 14 are presented in Fig. 1. Further analysis of the bifactor models excluding the Awareness subscale (Model 14 for DERS-SF and Model 19 for DERS-18) showed ECV values of 0.65 and 0.64, respectively, and ω_H_ values of 0.57 and 0.59. The ω_HS_ values for the subscales indicated that Clarity (0.49 and 0.51), Goals (0.62 and 0.61), and Impulse (0.54 and 0.50) exceeded or marginally met the threshold of 0.50. In contrast, the Nonacceptance subscale demonstrated relatively low ω_HS_ values, and the Strategies subscale showed the lowest values, of 0.16 in Model 14 and 0.17 in Model 19.

Comparing the five- and six-factor models, the five-factor models generally performed better than their corresponding six-factor counterparts, with lower RMSEA and SRMR values and a higher CFI value (Table 3). The modified five-factor model showed marginally acceptable fit for the DERS-SF (Model 15, *χ*^*2*^ = 867.522 [*df* = 122]; RMSEA = 0.070; CFI = 0.945; SRMR = 0.057) and relatively poor fit for DERS-18 (Model 20, *χ*^*2*^ = 1013.552 [*df* = 122]; RMSEA = 0.077; CFI = 0.937; SRMR = 0.062).

#### Internal consistency

The internal consistency of all subscales of the DERS-SF was within an acceptable range, with all Cronbach’s α and McDonald’s ω_S_ values greater than 0.70. However, the internal consistency of the Awareness subscale was slightly lower than that of the other subscales. The Cronbach’s α for the Awareness subscale was 0.73 and McDonald’s ω_S_ was 0.76, while the Cronbach’s α for the other subscales ranged from 0.77 to 0.92 and McDonald’s ω_S_ ranged from 0.78 to 0.92. In addition, the overall internal consistency was slightly higher when the Awareness scale was removed (Cronbach’s α and McDonald’s ω = 0.93) than when all six subscales were included (Cronbach’s α and McDonald's ω = 0.91). The internal consistency of the DERS-18 subscales was similar to that of the DERS-SF. The internal consistency of all other subscales ranged from 0.79 to 0.92 for Cronbach’s α and from 0.80 to 0.92 for McDonald’s ω_S_; however, the Awareness subscale had a Cronbach’s α of 0.74 and McDonald’s ω_S_ of 0.77. Overall internal consistency was higher when the Awareness subscale was removed (Cronbach’s α and McDonald’s ω = 0.93) than when it was included (Cronbach’s α and McDonald’s ω = 0.91).

#### Convergent validity

**Fig. 2:**
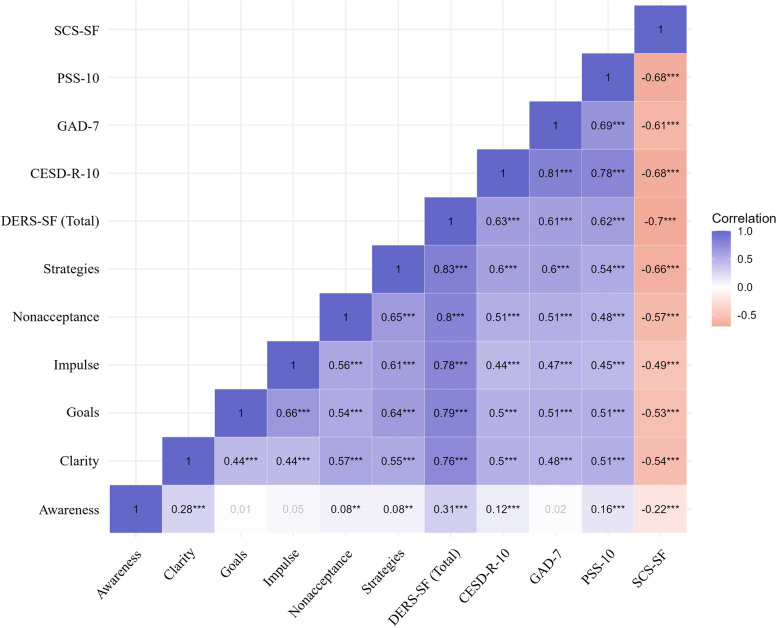
Correlations of DERS-SF scores with measures of depression, anxiety, perceived stress, and self-compassion. Note. DERS, Difficulties in Emotion Regulation Scale; SF, Short Form; CESD-R-10, 10-item Center for Epidemiologic Studies Depression Revised, GAD-7, 7-item Generalized Anxiety Disorder; PSS-10, Perceived Stress Scale, 10-item version; SCS-SF, Self-Compassion Scale-Short Form; ^***^ *P* < 0.001

**Fig. 3:**
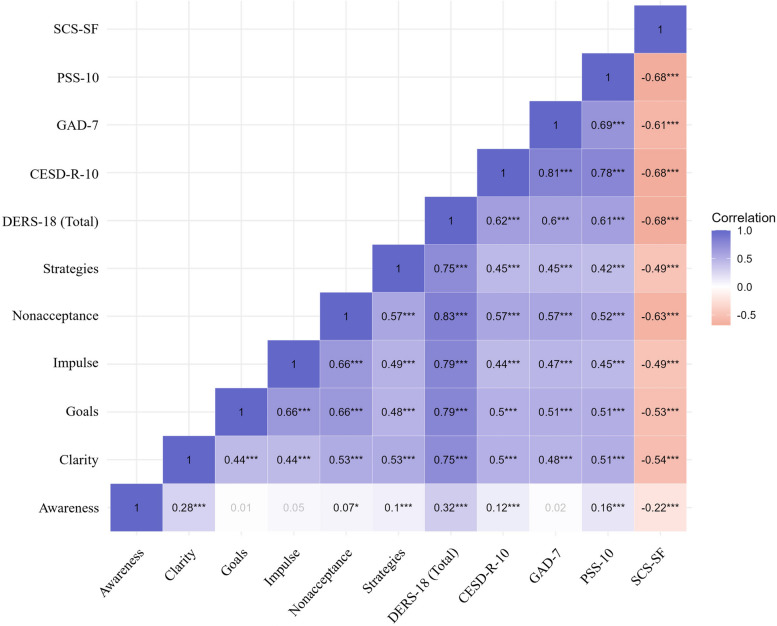
Correlations of DERS-18 scores with measures of depression, anxiety, perceived stress, and self-compassion. Note*.* DERS, Difficulties in Emotion Regulation Scale; CESD-R-10, 10-item Center for Epidemiologic Studies Depression Revised, GAD-7, 7-item Generalized Anxiety Disorder; PSS-10, Perceived Stress Scale, 10-item version; SCS-SF, Self-Compassion Scale-Short Form; ^***^ *P* < 0.001

The results from Pearson’s correlation analysis, summarized in Figs. 2 and 3, depict the relationships of the DERS-SF and DERS-18 scores with the CESD-R-10, GAD-7, PSS-10, and SCS-SF.

The total scores of both DERS-SF and DERS-18 showed strong positive correlations with measures of depression (CESD-R-10), anxiety (GAD-7), and perceived stress (PSS-10) (*r* = 0.60 to 0.63, all *P* < 0.001). In addition, they were negatively correlated with SCS-SF (*r* = –0.7 in DERS-SF, *r* = –0.68 in DERS-18, all *P* < 0.001). All subscales of both brief versions, except for the Awareness subscale, exhibited significant positive correlations with depression, anxiety, and stress (*r* = 0.42 to 0.60, all *P* < 0.001) and significant negative correlations with self-compassion (*r* = –0.66 to –0.49, all *P* < 0.001).

The Awareness subscale did not show significant correlations with anxiety in both brief versions (*P* = 0.41 in DERS-SF and DERS-18). Additionally, the Awareness subscale exhibited weak correlations with measures of depression (*r* = 0.12 in DERS-SF and DERS-18, all *P* < 0.001), perceived stress (*r* = 0.16 in DERS-SF and DERS-18, all *P* < 0.001), and self-compassion (*r* = –0.22 in DERS-SF and DERS-18, *P* < 0.001).

## Discussion

This study evaluated the latent factor structures and psychometric properties of brief versions of the DERS—DERS-SF, DERS-18, and DERS-16—within large community samples of Korean adults. The DERS-SF and DERS-18, which excluded the Awareness subscale, exhibited good reliability and fit indices for the internal structure of the questionnaires, while the DERS-16 exhibited inadequate fit indices. We also found that the DERS-SF and DERS-18 scores were closely related to indicators of psychological distress (i.e., depression, anxiety, and stress), supporting their adequate convergent validity. Furthermore, a bifactor model for both the DERS-SF and DERS-18, excluding the Awareness subscale, demonstrated the best fit with the data. Our findings suggest that the DERS-SF and DERS-18, with the bifactor model without the Awareness subscale, are efficient measures for assessing emotion dysregulation in the general population.

We conducted a series of CFAs to evaluate the factor structures of the brief versions of the DERS and compared various competing solutions. The DERS-SF and DERS-18 exhibited satisfactory fit indices (i.e., RMSEA, CFI, and SRMR), unlike the DERS-16. The superior model fits observed for the DERS-SF and DERS-18 are consistent with previous research on both community [39] and clinical [41] populations in the United States. Our findings also revealed that both the bifactor model excluding the Awareness subscale and the bifactor model including the Awareness subscale had acceptable model fit, with the former showing a slightly better fit for both the DERS-SF and DERS-18. Notably, the goodness-of-fit indices of the DERS-SF and DERS-18 did not distinctly favor one model over another.

Recent research has consistently indicated that the bifactor structure offers the best fit for the original DERS [22, 29], DERS-SF [28], and DERS-18 [30]. This study, which examined three brief versions of the DERS in a sizeable general population, further confirms the suitability of the bifactor models of the DERS-SF and DERS-18. While the ECV and ω_H_ values of bifactor models, excluding the Awareness subscale, suggest that the general factor accounts for a substantial portion of the variance, they do not fully support unidimensionality [58]. This highlights the significant contribution of specific factors to overall variance, justifying the interpretation and use of subscales. These findings emphasize the multidimensional nature of the DERS and underscore the importance of considering both a general factor and five specific factors to elucidate the meaning of the scores for the brief versions of the DERS. Reflecting the factor structure of the original DERS, which was designed to provide a multidimensional assessment of emotion dysregulation [18], its brief versions have been found to measure both unidimensional and multidimensional aspects of emotional dysregulation, providing a comprehensive explanation of difficulties with emotion regulation. Profiling specific factors of the emotion dysregulation measured by brief versions of the DERS can further clarify the nature of an individual’s emotion processing problems, enabling explicit recommendations for clinical intervention targets.

All three brief versions of the DERS demonstrated excellent overall internal consistency, with all values of Cronbach’s α and McDonald’s ω exceeding 0.9. These results are consistent with a previous study conducted on young adults in the United States [39]. Consistent with prior studies [28, 30], we observed a lack of reliability estimates for the Awareness subscale in both the DERS-SF and DERS-18. This subscale exhibited much lower correlations with the total score and other subscales, implying its divergence in measuring difficulties in emotion regulation compared with the remaining subscales. The Awareness subscale may represent a construct distinct from emotion dysregulation as measured by the other subscales of the DERS.

Significant disparities in the psychometric properties of the Awareness subscale are likely attributable to method effects. Method effects involve tendencies to respond to survey items based on criteria unrelated to the intended content being measured, leading to irrelevant systematic variance not aligned with the study or concept under examination [59]. The Awareness subscale is unique in that it exclusively comprises reverse-scored items. Previous literature has indicated potential measurement issues associated with the inclusion of reverse items [60], such as reduced internal consistency [61, 62] and poorer model fit [63]. This underscores the impact of item configurations in the Awareness subscale on the overall psychometric properties of the DERS.

Another explanation may be that emotional awareness operates at a different stage in the emotion regulation processes [64]. A previous study [31] noted that emotional awareness and clarity may forego the actual use of emotion regulation strategies and the presence of emotional awareness, linked to the cognitive aspects of emotional experiences, does not guarantee the use of adaptive emotion regulation strategies. This aligns with Gratz and Roemer’s original framework [18], which conceptualized Awareness and Clarity as a single construct. In this study, modified five-factor models of the DERS-SF and DERS-18, which combined Awareness and Clarity items into a single construct with a method factor, demonstrated marginal fit. These findings suggest that Awareness and Clarity may represent a unified dimension. Future research should further outline the boundaries and overlaps between these constructs and explore their potential role as core components of emotion regulation or as elements of broader constructs, such as emotional understanding or identification [29, 33, 34].

The results of the correlation analyses revealed significant relationships between self-reported emotion dysregulation, as measured by the brief versions of the DERS, and other clinical measures including depression, anxiety, perceived stress, and self-compassion. The directions of the correlations were aligned with our expectations, confirming the utility of the DERS-SF and DERS-18 in gauging emotion dysregulation. Individuals with elevated total scores on the DERS-SF and DERS-18 were more likely to experience symptoms of depression and anxiety. This is consistent with a previous meta-analytic study illustrating the associations of emotion regulation capacities with symptoms of depression and anxiety [65]. The significant correlations of the brief versions of the DERS with subjective stress and self-compassion support the perspective that emotion dysregulation reflects individuals’ vulnerability, characterized by a lack of coping strategies and reduced resources for managing adverse events, potentially contributing to general distress.

The findings not only support the convergent validity of the brief versions of the DERS, but also emphasize the importance of further investigation into the unique correlates of their subscales. In this study, all subscales, except the Awareness, exhibited similar patterns of correlation with depression, anxiety, perceived stress, and self-compassion. If these subscales indeed capture related yet distinct dimensions of emotion dysregulation, they would demonstrate unique associations with specific constructs of emotion regulation (e.g., experiential, behavioral, and physiological responses [66]) or clinical outcomes [67]. Previous studies have reported that subscales of the DERS exhibit distinct patterns of association based on psychopathology comorbidity [26], clinical severity [22], and suicide risk [29], supporting the notion that these subscales reflect unique dimensions of emotion dysregulation. Future research should explore these patterns to clarify the unique contributions of each subscale.

Several limitations of the study should be considered. First, the study exclusively involved community adults, and the absence of test–retest reliability assessments for the brief versions of the DERS underscores the need for future research. To enhance generalizability, longitudinal investigations should encompass both community and clinical samples to clarify the stability of measures over time and across various participants’ characteristics for each brief version of the DERS. Second, online surveys are characterized by increased flexibility in initiation and persistence of participation. Thus, individuals with a particular interest in the survey topic may have been more engaged in completing the entire survey, introducing potential biases into the data. Additionally, while we manually reviewed the data for response patterns indicative of invalid responding (e.g., selecting the same option for all items), the absence of formal attention check items or validity scales in the study design remains a limitation. This may have allowed undetected invalid responses to contribute to error variance in our analyses. Lastly, the Strategies subscale exhibited notably low ω_HS_ values (0.16 for the DERS-SF and 0.17 for the DERS-18), indicating that only a minimal proportion of its variance could be attributed to a specific factor. This finding is consistent with prior research [29], highlighting the need for further investigation into the validity and interpretability of this subscale.

To our knowledge, this study is the first examination of the competing factor structures and psychometric properties of the brief versions of the DERS simultaneously within the same large community samples. In summary, our findings demonstrate that both the DERS-SF and DERS-18 function as robust measures of emotion dysregulation with fewer items than initially assumed in the original version. In addition, the exclusion of items from the Awareness subscale in brief versions of the DERS enhances reliability and validity in assessing emotion dysregulation. The DERS-SF and DERS 18, represented by a bifactor model, showed satisfactory reliability and validity. This streamlined approach improves the identification of individuals at risk of various mental health challenges, enabling targeted interventions and personalized treatment strategies tailored to specific emotion dysregulation profiles.

## Data Availability

The data that support the findings of this study are not publicly available due to ethical considerations: Participants did not provide written consent for their data to be publicly shared. Instead, de-identified participant data will be made available upon reasonable request from the corresponding author.
